# High-resolution magnetic resonance imaging of intracranial vessel walls: Comparison of 3D T1-weighted turbo spin echo with or without DANTE or iMSDE

**DOI:** 10.1371/journal.pone.0220603

**Published:** 2019-08-06

**Authors:** Se Jin Cho, Seung Chai Jung, Chong Hyun Suh, Jung Bin Lee, Donghyun Kim

**Affiliations:** Department of Radiology and Research Institute of Radiology, University of Ulsan College of Medicine, Asan Medical Center, Songpa-Gu, Seoul, Republic of Korea; Universitatsklinikum Freiburg, GERMANY

## Abstract

**Background:**

The black-blood (BB) technique was developed to suppress the signal from blood and cerebrospinal fluid (CSF) to provide improved depiction of vessel walls.

**Purpose:**

The aim was to compare three-dimensional turbo spin echo T1-weighted imaging (3D TSE T1WI) with or without two BB techniques (delay alternating with nutation for tailored excitation [DANTE], and improved motion-sensitized driven equilibrium [iMSDE]) for high-resolution magnetic resonance imaging (HR-MRI) of the vessel walls of intracranial arteries.

**Study type:**

Prospective.

**Population:**

Fourteen healthy volunteers who underwent 3D T1WI for examination of intracranial vessel walls.

**Field strength/Sequence:**

3 Tesla, 3D TSE T1WI (SPACE and BrainVIEW) and BB (DANTE and iMSDE).

**Assessment:**

SPACE with or without DANTE, and BrainVIEW with or without iMSDE, were acquired in each subject. Two neuroradiologists independently assessed image quality, vessel wall delineation, BB effect, CSF, and acceptability using visual scoring systems, and measured signal-to-noise ratio (SNR) and contrast-to-noise ratio (CNR) in vessel walls, lumen, and CSF, while blinded to the presence and type of BB technique used.

**Statistical tests:**

Repeated measures ANOVA or Friedman tests were performed for the comparisons, followed by Bonferroni correction.

**Results:**

The 3T T1WI sequences without BB are significantly superior in vessel wall delineation (P = 0.001). Black CSF scores were lower in SPACE with DANTE than SPACE without DANTE, and in BrainVIEW without iMSDE than SPACE without DANTE (P < 0.001). However, there were no significant differences in BB effect, image quality, and acceptability between the four 3D T1WI sequences (p > .05). The SNR_Vessel wall_, CNR_Wall-Lumen_, and CNR_Wall-CSF_ were higher (all p < .001) on SPACE with and without DANTE than on BrainVIEW with and without iMSDE. SNR_Lumen_ were higher (all p < .001) on BrainVIEW with and without iMSDE than on SPACE with and without DANTE. SNRCSF was higher (all p < .001) on BrainVIEW with iMSDE than on SPACE with DANTE.

**Data conclusion:**

Both 3D TSE T1WI sequences were acceptable for intracranial vessel wall evaluation, with or without BB techniques. Therefore, BB techniques may not necessarily be required with 3D TSE T1WI with a long ETL and TR (below 1160 ms).

## Introduction

High-resolution magnetic resonance imaging (HR-MRI) of intracranial vessel walls can depict the vessel walls directly. This can narrow the differential diagnosis and improve diagnostic accuracy for various intracranial artery diseases, beyond that achievable from the indirect information obtained from luminal angiography [1–6].

Three-dimensional (3D) HR-MRI has been widely used for vessel wall imaging, offering the advantages of a large coverage volume and multi-planar imaging planes with whole coverage of intracranial arteries [5–9]. 3D turbo spin echo (3D TSE) imaging sequences with variable flip angle (FA) have been widely used for 3D HR-MRI vessel wall evaluation, with these sequences including SPACE (Siemens Healthcare), BrainVIEW (Philips Medical System), and CUBE(GE health care).

Various black-blood (BB) imaging techniques have been used to suppress blood and cerebrospinal fluid (CSF) signal in 3D TSE T1-weighted (T1WI) vessel wall imaging [10, 11], including iMSDE (improved motion-sensitized driven equilibrium) and DANTE (delay alternating with nutation for tailored excitation). These two BB methods have been regarded modalities with better performance in evaluation of intracranial walls than 3D TSE alone [8, 12–14]. However, 3D TSE T1WI does have an intrinsic BB effect due to the dephasing of moving blood spins, even without the addition of an intentional BB technique [15, 16]. Furthermore, deliberate BB techniques can cause signal loss [9, 12, 13]. The highest differentiation of vessel walls from CSF and brain parenchyma in 3D TSE T1WI having been achieved with a TR of 1160 ms [17]. The intrinsic BB effect in 3D TSE T1WI can also be enhanced by longer echo train lengths (ETLs) and refocusing pulses with low FAs [16]. Therefore, 3D TSE T1WI may be tuned to present a usable BB and black-CSF effect by selection of appropriate TR while keeping acquisition time clinically reasonable.

To our knowledge, comparisons between SPACE and BrainVIEW, and between DANTE and iMSDE, for intracranial vessel wall evaluation have not been reported. Therefore, in this study, we aimed to compare two 3D TSE T1WI sequences (SPACE and BrainVIEW), with or without two BB techniques (DANTE and iMSDE), in intracranial vessel wall evaluation.

## Materials and methods

### Subjects

Institutional review board approval in Asan Medical Center for this study and informed consent from each subject were obtained (2017–0902). From September 2017 to November 2017, 14 healthy volunteers were enrolled for the current study and they underwent 3D TSE T1WI for vessel wall imaging of intracranial arteries at a single institute. The 14 subjects had a mean age of 56.9 years (age range, 33–67 years), seven were men (mean age, 58.6 years; age range, 40–67 years), and seven were women (mean age, 55.3 years; age range, 33–65 years).

### MRI protocol

All HR-MRI examinations were performed using 3-Tesla systems (Skyra, Siemens Healthcare, Erlangen, Germany, with a 64-channel head and neck coil; Ingenia CX, Philips Medical Systems, Best, the Netherlands, with a 32-channel head surface coil). Four 3D T1WI MRI sequences were acquired from each subject using SPACE (Siemens Healthcare) and BrainVIEW (Philips Medical Systems) acquisitions with and without the corresponding BB technique. The imaging parameters for T1W SPACE included the following: TR, 900 ms; echo time (TE), 15 ms; FA, 120°; ETL, 70; matrix, 320 × 320 × 333; field of view, 192 × 192 × 200 mm; voxel size, 0.6 × 0.6 × 0.6 mm; CAIPIRINHA acceleration factor, 2; acquisition time, 8 min 12 s; and fat saturation with or without DANTE [18]. The imaging parameters for T1WI BrainVIEW included the following: TR, 900 ms; TE, 16.8 ms; FA, 90°; ETL, 48; matrix, 320 × 320 × 333; field of view, 192 × 192 × 200 mm; voxel size, 0.6 × 0.6 × 0.6 mm; SENSE acceleration factor, 2; acquisition time, 9 min 18 s; and fat saturation with or without iMSDE [19].

### Analysis of MRI images

Two radiologists (XXX and XXX; both with 5 years of experience in neuroradiology), who were blinded to the subject details and presence and type of BB technique, each independently performed qualitative analysis using visual scoring systems (image quality, vessel wall delineation, BB and black-CSF signal, and acceptability) and quantitative measurements (signal-to-noise ratio [SNR] and CNR in vessel walls, lumen, and CSF) of the images. The qualitative and quantitative analyses were performed in the same location of mid-M1 and mid- M2 segments of middle cerebral artery (MCA), distal basilar artery (BA), and terminal internal carotid artery (ICA). Additionally, M2/3 junction of MCA was qualitatively analyzed. For the qualitative analysis, images of each MRI sequence were rated using four-point visual scoring systems (0: poor; 3: excellent) [20]. These visual scoring systems were defined as follows: image quality: 0, poor image quality with large artifact; 1, moderate image quality with moderate artifact; 2, good image quality with slight artifact; and 3, excellent image quality without artifact. Vessel wall delineation: 0, less than 50% of the vessel wall visible; 1, more than 50% of the vessel wall visible; 2, vessel wall delineated with adequate signal and contrast to the lumen and CSF; 3, vessel wall delineated with excellent signal and sharp contrast to the lumen and CSF [20]. BB performance: 0, less than a quarter of the target lumen dimension visible with black signal intensity (SI); 1, from a quarter to a half of the target lumen dimension visible with black SI; 2, from a half to three-quarters of the target lumen dimension visible with black SI; 3, more than three-quarters of the target lumen dimension visible with dark SI. The CSF was also scored using a four-point scale divided into two parts as follows. (Part a) In terms of the compartment homogeneity measured on a medial sagittal image plane: 0, heterogeneous; 1, one or two CSF ventricular or cisternal space visible with non-black SI; 2, all CSF compartments visible with black SI. (Part b) In terms of the SI in comparison with the vitreous humor of the eyeball measured on an axial image: 0, high SI compared with the vitreous humor; 1, similar or lower SI than the vitreous humor. The four-point total CSF score was then derived as the sum of parts a and b. Finally, clinical acceptability considering vessel wall delineation, black blood and CSF performance, and image quality were defined as follows: 0, definitely unacceptable; 1, unacceptable; 2, acceptable; and 3, definitely acceptable. The acceptability was assessed based on the whole images and covered intracranial arteries.

For the quantitative analysis, SNR, and CNR were measured. The pooled data irrespective of the vessel location was used for the analysis. SNR_wall_ was calculated as follows: SNR = 0.695 × (SI)/(noise), with noise being measured as the standard deviation of the white matter signal (region of interest area > 200 mm^2^) [21]. CNR_wall-lumen_ and CNR_csf-lumen_ were calculated as CNR_A-B_ = SNR_A_−SNR_B_ [20].

### Statistical analysis

MedCalc for Windows Version 1.1.1.0 (MedCalc Software, Mariakerke, Belgium) was used for the analyses. The comparison was performed between SPACE with DANTE and without DANTE, Brainview with iMSDE and without iMSDE, SPACE without DANTE and Brainview without iMSDE, SPACE with DANTE and Brainview with iMSDE. The Kolmogorov-Smirnov test was used to determine whether the continuous variables were normally distributed. Following the results of the Kolmogorov-Smirnov test, either a repeated measures ANOVA test or Friedman tests were performed for the comparisons. Cochran’s Q test was performed for the comparison proportions of acceptability. Post-hoc tests were performed using Bonferroni correction. A p-value < .05 was considered to indicate a statistically significant difference.

Interobserver agreement was assessed using intraclass correlation coefficient (ICC) and weighted kappa for continuous variables and nominal variables, respectively. The strength of the interobserver agreement ICCs and weighted kappa were categorized as follows: < 0.20, poor; 0.21–0.40, fair; 0.41–0.60, moderate; 0.61–0.80, good; 0.81–1.00, excellent [22].

## Results

### Visual scoring systems

**Fig 1:**
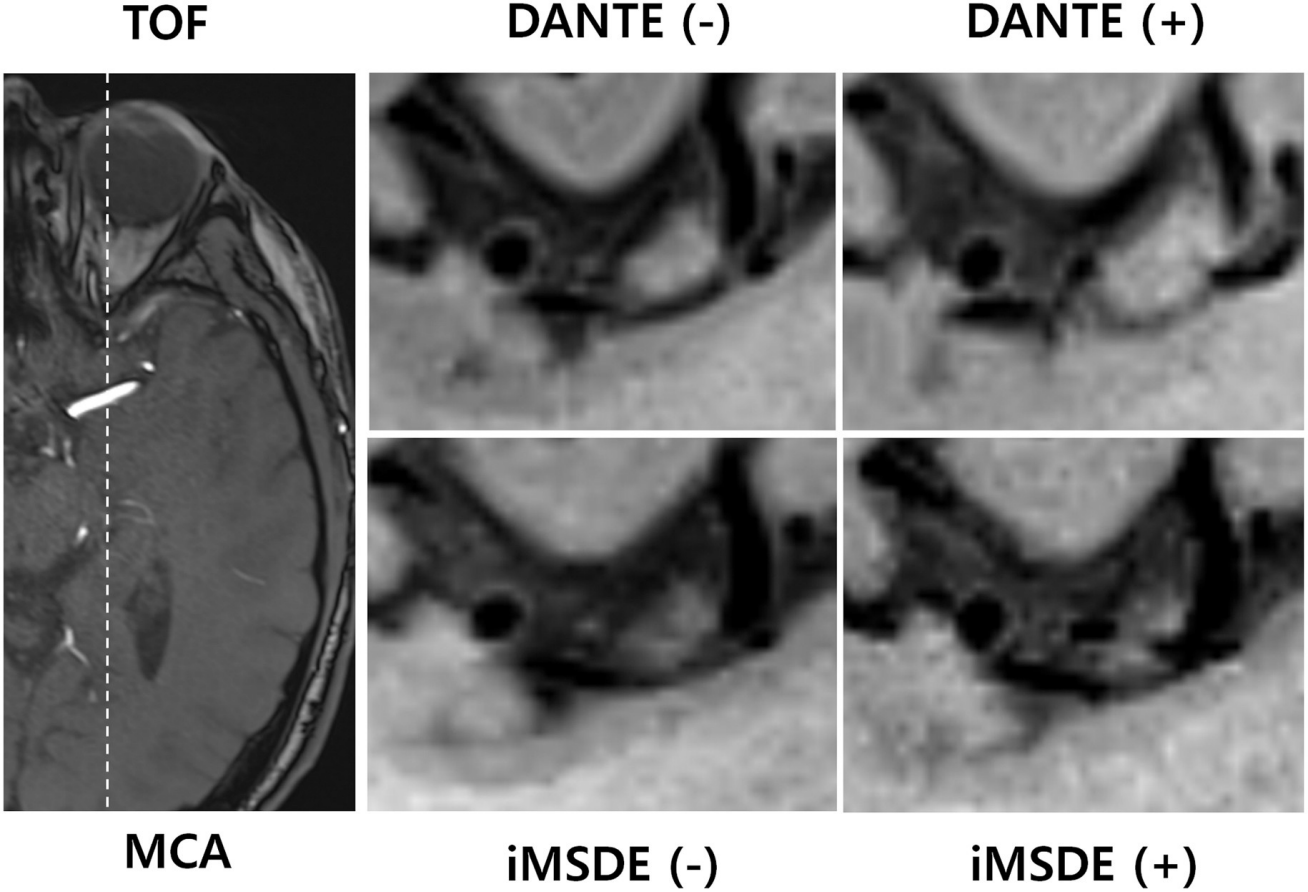
Sagittal images in left M1 segment of middle cranial artery were obtained from a 37 year-old male patient. The overall image quality, vessel wall delineation, black blood, and black CSF for four different sequences shows clinically acceptable. iMSDE = improved motion-sensitized driven equilibrium, DANTE = delay alternating with nutation for tailored excitation.

**Fig 2:**
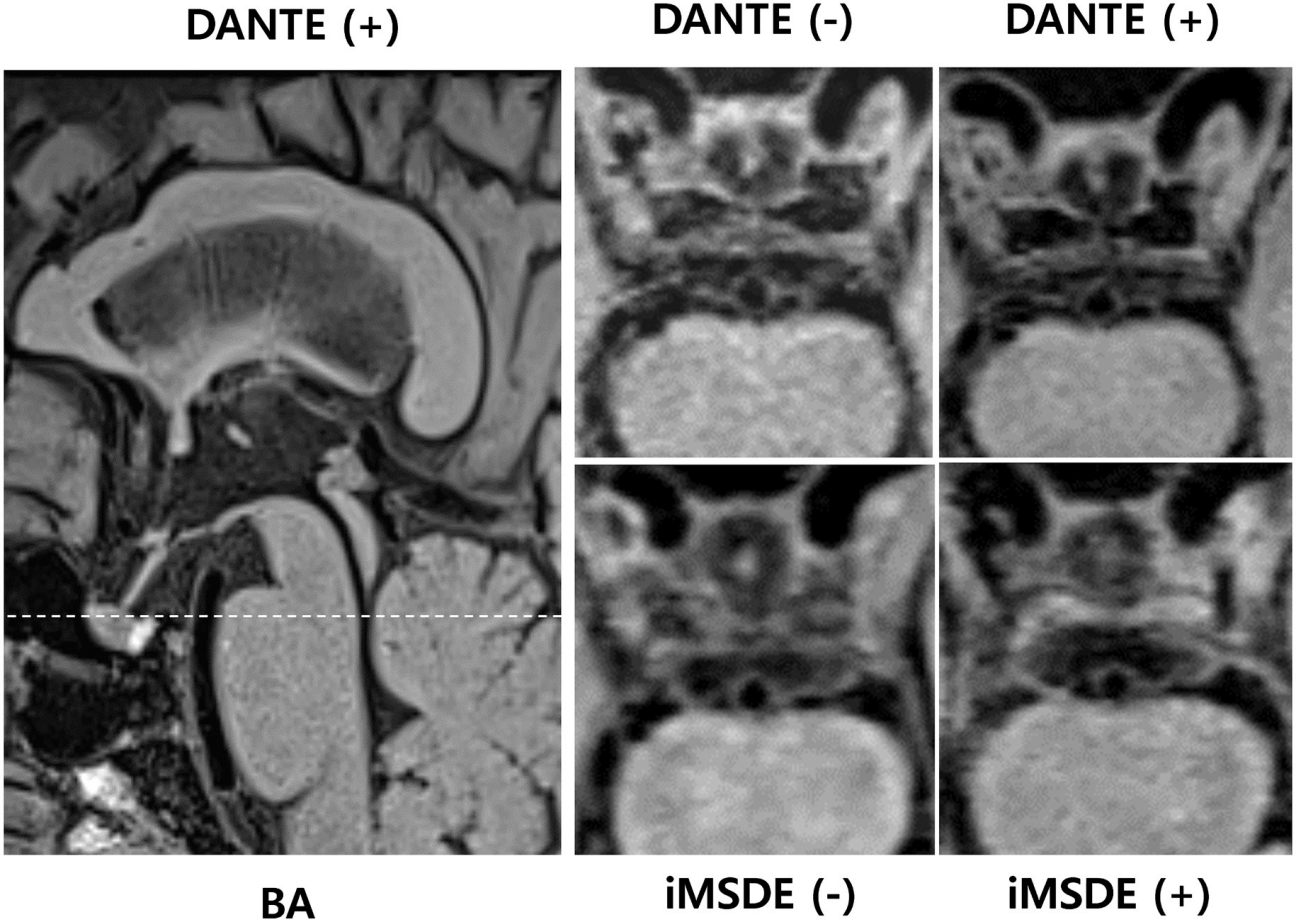
Axial images in basilar artery were obtained from a 67 years-old male patient. The overall image quality, vessel wall delineation, black blood, and black CSF for four different sequences shows clinically acceptable. iMSDE = improved motion-sensitized driven equilibrium, DANTE = delay alternating with nutation for tailored excitation.

The 3T T1WI sequences without BB are significant superior in vessel wall delineation (P = 0.001). Black CSF scores were lower in SPACE with DANTE than SPACE without DANTE, and in BrainVIEW without iMSDE than SPACE without DANTE (P < 0.001). However, there were no significant differences in BB effect, image quality, and acceptability between the four 3D T1WI sequences (p > .05) (Table 1). Figs 1 and 2 shows representative coronal and axial images.

**Table 1:** Comparisons of visual scoring system results between 3D TSE T1WI with or without iMSDE and DANTE.

Category	3D T1WI without iMSDE	3D T1WI without DANTE	3D T1WI with iMSDE	3D T1WI with DANTE	P-value	Mean Difference	95% CI
**Vessel wall delineation**	2.96	2.94	2.80	2.83	0.001 †, ‡	0.138	0.042 ~ 0.23
**Black blood**	2.93	2.96	2.96	2.96	1	0.03	-0.048 ~ 0.11
**Black CSF**	2.39	2.93	2.36	2.54	< 0.001*,‡	0.536	0.26 ~ 0.81
**Image quality**	3	3	3	3	NA	NA	NA
**Acceptability**	2.79	2.93	2.93	3	0.26	0.143	-0.049 ~ 0.34

T1WI = T1-weighted imaging, iMSDE = improved motion-sensitized driven equilibrium, DANTE = delay alternating with nutation for tailored excitation. The symbols (* †, and ‡) represent p-values between between T1WI without iMSDE and T1WI without DANTE (*), T1WI without iMSDE and T1WI with iMSDE (†), and T1WI without DANTE and T1WI with DANTE (‡). Statistical significance was demonstrated by Bonferroni corrected post-hoc tests.

### SNR and CNR

The SNR_Vessel wall_, CNR_Wall-Lumen_, and CNR_Wall-CSF_ were higher (all p < .001) on SPACE with and without DANTE than on BrainVIEW with and without iMSDE. SNR_Lumen_ were higher (all p < .001) on BrainVIEW with and without iMSDE than on SPACE with and without DANTE. SNR_CSF_ was higher (all p < .001) on BrainVIEW with iMSDE than on SPACE with DANTE and (Table 2). SNR and CNR show significant decrease from center to peripheral location in the spatial locations (M1, M2, BA, ICA) (p < 0.05) (S1 Table).

**Table 2:** Comparisons of SNR and CNR between 3D TSE T1WI with or without iMSDE and DANTE.

	3D T1WI without iMSDE	3D T1WI without DANTE	3D T1WI with iMSDE	3D T1WI with DANTE	P-value	Mean Difference	95% CI
**SNR** Vessel wall	11.21	14.02	8.95	11	<0.001*, †, ‡, §	3.02	2.39 ~ 3.65
**SNR** Lumen	3.04	1.14	2.31	1.28	<0.001*, †, ‡, §	1.9	1.67 ~ 2.14
**SNR** CSF	7.51	6.64	7.25	6.48	<0.003§	1.03	0.28 ~ 1.77
**CNR** Wall-Lumen	8.17	12.88	6.71	9.72	<0.001*, †, ‡, §	4.71	3.8 ~ 5.6
**CNR** Wall-CSF	3.73	7.37	1.8	4.56	<0.001*, †, ‡, §	3.61	2.83 ~ 4.39

T1WI = T1-weighted imaging, iMSDE = improved Motion-Sensitized Driven Equilibrium, DANTE = Delay Alternating with Nutation for Tailored Excitation, SNR = signal-to-noise ratio, CNR = contrast-to-noise ratio. The symbols (*, †, ‡, and §) represent p-values between T1WI without iMSDE and T1WI without DANTE (*), T1WI without iMSDE and T1WI with iMSDE (†), T1WI without DANTE and T1WI with DANTE (‡), and T1WI with iMSDE and T1WI with DANTE (§). Statistical significance was demonstrated by Bonferroni corrected post-hoc tests.

### Interobserver agreement

Moderate to excellent interobserver agreements (kappa, 0.627 to 1.000) were observed in all the qualitative analyses using visual scoring systems. Good to excellent interobserver agreements were observed (ICCs, 0.898 to 0.972) in the quantitative analyses of SNR and CNR (Table 3).

**Table 3:** Interobservor agreements between two radiologists in qualitative and quantitative analyses.

Variables	Weighted Kappa * or ICC †	95% CI	Category
**Qualitative analysis**			
**Vessel wall delineation**	0.91*	0.85, 0.97	Excellent
**Black blood**	0.77*	0.68, 0.87	Good
**Black CSF**	0.86*	0.72, 0.99	Excellent
**Image quality**	0.6*	0.42, 0.79	Moderate
**Acceptability**	0.78*	0.49, 1	Good
**Quantitative analysis**			
**SNR**	0.97†	0.96, 0.97	Excellent
**CNR**	0.96†	0.9, 0.96	Excellent

The symbols * represent value of Weighted Kappa, and

^†^ of ICC.

CI = confidence interval, ICC = Intraclass correlation coefficient

## Discussion

This study shows that all four 3D TSE T1WI sequences provided acceptable results in the vessel wall of intracranial arteries, even those without a specific BB technique. The 3D TSE T1WI without BB were superior than The 3D TSE T1WI with BB in vessel wall delineation (P = .001) and SNR_Vessel wall_ (P < 0.001). Therefore, BB may not necessarily be required for 3D TSE T1WI with a long ETL and appropriate TR. In addition, SPACE and Brainview were comparable in the whole visual scoring system except in black CSF. Therefore, both SPACE and Brainview may be acceptable as intracranial vessel wall MRI.

In 3D TSE T1WI, the intrinsic BB effect can be enhanced by the use of longer ETLs and refocusing pulses with low FAs [16]. The differentiation of vessel walls from CSF and brain parenchyma at the intracranial arterial can be enhanced by the use of a TR of around 1000 ms [17], even though a longer TR lengthens the scan time. Previous studies reported that 3D TSE T1WI with BB techniques produced a significantly superior BB effect to 3D TSE T1WI without specific BB contrast [8, 12–14]. However, these studies used 3D TSE T1WI with TRs far from 1000 ms (2000, 770, and 400 ms), and relatively short ETLs (38, 31, and 17) [9, 12, 13]. In this study, TRs of 900 ms and ETLs of 70 or 48 were used for all sequences, which may have led to the similar vessel wall delineation and BB and black-CSF effects, irrespective of whether a specific BB contrast was used. We also found that the recently introduced BB techniques of iMSDE and DANTE still resulted in significant reductions in SNR and CNR.

To our knowledge, no previous report has compared SPACE with or without DANTE, and BrainVIEW with or without iMSDE, for imaging of intracranial arterial vessel walls. DANTE suppresses the signal from moving spins with a series of low FA nonselective pulses interleaved with gradient pulses with short TRs. Moving spins in the vessel flow are spoiled by the applied gradient [9]. The iMSDE technique is an improvement on MSDE, which uses motion-sensitized gradients to dephase all moving spins in the vessel walls. The MSDE sequence consists of two 90° excitation pulses and a 180° refocusing pulse with unipolar motion-sensitized and spoiled gradients, while iMSDE consists of two 90° excitation pulses and two 180° refocusing pulses with bipolar motion-sensitized and spoiled gradients. iMSDE achieved improved SNR and CNR compared with MSDE [14]. In this study, both DANTE and iMSDE caused a reduction in SNR and CNR, except for SNR_Lumen_, even though there was no significant reduction in the visual scoring systems. Furthermore, there were significant differences in SNR and CNR between SPACE with or without DANTE and BrainVIEW with or without iMSDE, even though there were no significant differences in the visual scoring systems except in black CSF. Therefore, SPACE and BrainVIEW, and DANTE and iMSDE, seem to be comparable 3D TSE T1WI and BB techniques, in the clinical application, which may be supported by the acceptability, and SPACE and Brainview without BB may be more appropriate.

This study has several limitations. First, only 14 healthy volunteers were enrolled in this study. Therefore, further study should also be evaluated on patients with intracranial arterial disease, as well as on larger sample size to overcome the lack of generality and underpowered analysis in this study. The imaging sequences should also be evaluated on patients with intracranial arterial disease, as well as on a larger sample size. In particular, subjects with fusiform aneurysmal or steno-occlusive lesions should be studied to determine the suitability of 3D TSE T1WI without BB. Second, the optimization for appropriate TR and ETL was based on the previous study and preliminary testing. However, more thorough optimization of TR and ETL are warranted. Third, the comparisons between the two different BB techniques were not performed with the same 3D TSE T1WI sequences and the same MR scanners and coils. However, we used the commercially released BB sequences from each vendor without making any changes, and the sequences and coils are inherent to each MR system and reflect what is commercially and clinically available. Therefore, our results should be directly applicable to the clinical field. Fourth, contrast-enhanced 3D TSE T1WI is also widely used to provide important information for vessel wall evaluation, but contrast enhancement was not used in this study involving normal volunteers. Finally, our results may have the lack of generalization and thus further study is necessary because this study was performed under the specific 3D TSE T1WI and BB techniques.

In conclusion, both 3D TSE T1WI sequences were acceptable for intracranial vessel wall evaluation, with or without BB techniques. Therefore, BB techniques may not necessarily be required with 3D TSE T1WI with a long ETL and TR (below 1160 ms to select appropriate TR while keeping acquisition time clinically reasonable). However, more thorough optimization of TR and ETL are warranted.

## Supporting information

S1 Table Comparisons of quantitative analyses of black blood performances between the vascular locations. (DOCX)
