# Supplementary material for: High-resolution magnetic resonance imaging of intracranial vessel walls: Comparison of 3D T1-weighted turbo spin echo with or without DANTE or iMSDE
Source: PLoS One. 2019 Aug 6;14(8):e0220603. doi: 10.1371/journal.pone.0220603 (PMC6684065; doi:10.1371/journal.pone.0220603)
Supplement: S1 Table — (DOCX) [file pone.0220603.s001.docx]

**Supplementary table 1.** Comparisons of quantitative analyses of black blood performances between the vascular locations.

| Category | Mid-M2 | Mid-M1 | Distal BA | Terminal ICA | P-value |
| --- | --- | --- | --- | --- | --- |
| SNR Lumen |  |  |  |  |  |
| T1 without DANTE | 0.67 | 1.20 | 1.46 | 1.29 | <0.05 *,†, ‡ |
| T1 with DANTE | 0.64 | 1.41 | 1.60 | 1.46 | <0.05 *,†, ‡ |
| T1 with iMSDE | 1.63 | 2.71 | 2.43 | 2.47 | <0.05 *,†, ‡ |
| T1 without iMSDE | 2.49 | 3.11 | 2.88 | 3.69 | <0.05 †, ‡ |
| CNR Wall-Lumen |  |  |  |  |  |
| T1 without DANTE | 9.29 | 12.25 | 11.42 | 18.55 | <0.05 *,†, ‡, ‖,¶ |
| T1 with DANTE | 7.17 | 9.04 | 7.98 | 14.7 | <0.05 *, ‡, ‖,¶ |
| T1 with iMSDE | 7.17 | 5.88 | 5.79 | 7.98 | <0.05 *,†, ‖,¶ |
| T1 without iMSDE | 7.63 | 6.49 | 7.24 | 11.31 | <0.05 ‡, ‖,¶ |

BA= basilar artery, CNR = contrast-to-noise ratio, DANTE = Delay Alternating with Nutation for Tailored Excitation, ICA= internal carotid artery, iMSDE = improved Motion-Sensitized Driven Equilibrium, mid-M1 = mid-M1 and mid- M2 segments of middle cerebral artery (MCA), mid-M2= mid- M2 segments of MCA, and SNR = signal-to-noise ratio.

The symbols (*, †, ‡, §, ‖, and ¶) represent p-values between mid M2 and mid M1 (*), mid M2 and distal BA (†), mid M2 and terminal ICA (‡), mid M1 and distal BA (§), mid M1 and terminal ICA (§), and between distal BA and terminal ICA (¶). Statistical significance was demonstrated by Bonferroni corrected post-hoc tests.
